# Investigation of using very high‐frequency ultrasound in the differential diagnosis of early‐stage pemphigus vulgaris vs seborrheic dermatitis

**DOI:** 10.1111/srt.12836

**Published:** 2020-01-13

**Authors:** Xiaofeng Zheng, Chao Wu, Hongzhong Jin, Jie Liu, Haimeng Wang

**Affiliations:** ^1^ Department of Dermatology Peking Union Medical College Hospital Beijing Union Medical College Chinese Academy of Medical Sciences Beijing China

**Keywords:** pemphigus vulgaris, seborrheic dermatitis, very high‐frequency ultrasound

## Abstract

**Background:**

Early‐stage pemphigus vulgaris (PV) often manifests as erythema alone. We hypothesized that very high‐frequency ultrasonography (VHFUS) features could simplify the differential diagnosis of early‐stage pemphigus vulgaris versus seborrheic dermatitis (SD).

**Materials and Methods:**

Fourteen patients with SD or early‐stage PV were recruited from our outpatient clinic during 2016‐2019. We used 50‐MHz VHFUS to image the erythema on the patients’ scalp, face, chest, and back and retrospectively evaluated their ultrasonographic features.

**Results:**

Very high‐frequency ultrasonography images of early‐stage PV showed enhanced epidermal echo (8/14, 57%), linear or oval intra‐epidermal hypoechoic/anechoic areas (12/14, 86%), linear anechoic areas at the dermal‐epidermal junction (14/14, 100%), reduced echo of superficial to whole dermis (9/14, 64%), and slightly increased dermal thickness (14/14, 100%). The intra‐epidermal hypoechoic/anechoic bands (100%) showed the greatest specificity. VHFUS images of SD showed enhanced epidermal echo (7/14, 50%), epidermal unevenness (7/14, 50%), linear anechoic bands at the dermal‐epidermal junction (8/14, 57%), reduced middle dermis echo (10/14, 71%), and slightly increased dermal thickness (10/14, 71%). The epidermal unevenness (100%) had the greatest specificity. There was a significant difference (*P* < .05) between early‐stage PV and SD regarding the proportion of linear intraepithelial hypoechoic/anechoic bands and linear anechoic areas at the dermal‐epidermal junction.

**Conclusions:**

Early‐stage PV and SD have relatively specific VHFUS erythematous manifestations. Intra‐epidermal hypoechoic/anechoic bands for early‐stage PV and epidermal unevenness for SD were most specific. VHFUS contributes to the differential diagnosis of PV and SD by highlighting their features, that is, intra‐epidermal hypoechoic/anechoic bands and linear anechoic areas at the dermal‐epidermal junction.

## 1 INTRODUCTION

Pemphigus vulgaris (PV) is an autoimmune, bullous, skin disease. Early clinical manifestations of the disease, characterized by multiple erythematous areas, erosion, and scabs on the head, face, chest, and back, are easily misdiagnosed before the appearance of the typical blisters, bullae, and mucosal damage. Seborrheic dermatitis (SD) is characterized by multiple erythematous areas and greasy scales or crusts on the head and face, chest, and back, similar to the clinical manifestations of PV in its early stage. Hence, the two are often confused in clinical practice and must be differentiated by histopathological and/or direct immunofluorescence examinations and other invasive tests, which are often difficult to implement.

Very high‐frequency ultrasound (VHFUS) is a high‐resolution, noninvasive, objective examination that has been used as an auxiliary means to evaluate superficial tumors and internal organs in the past. In recent years, VHFUS has proved to have unique value in the identification of inflammatory lesions of the skin and subcutaneous tissues, such as scleroderma^1^ and psoriasis.^2^ The use of VHFUS for evaluating autoimmune bullous skin diseases has rarely been reported. We therefore used VHFUS to study the characteristics of erythema in patients with PV at an early stage and those with SD and then explored the value of VHFUS in their differential diagnosis.

## 2 PATIENTS AND METHODS

### 2.1 Patients

This retrospective study was conducted from June 2016 to April 2019, during which time we enrolled 14 patients with early‐stage PV and 14 with SD who were recruited from the outpatient clinic of Peking Union Medical College Hospital. The diagnosis was confirmed in each case based on their clinical manifestations and histopathological or immunofluorescence examination. Patients with PV were all in the early stage of the disease course and mainly presented with erythema, erosion, and scabs on the head, face, and/or chest and back. None had yet developed the typical blisters or bullae. The patients with SD presented with erythema and greasy scales on the head, face, and/or chest and back.

For the PV patients, the male:female ratio was 1:1.8, and the age range was 27‐73 years (mean 50 ± 14 years). Samples of the lesions were collected from the head and face in three cases and the trunk in 11 cases. For SD patients, the male:female ratio was 1:2.5, and the age range was 22‐54 years (mean 44 ± 10 years). Samples from their lesions were collected from the head and face in nine cases and the trunk in five cases.

### 2.2 Acquisition and evaluation of ultrasonic images

The VHFUS instrument was produced by Tianjin Minda Company. Ultrasonic images of patients with new erythematous areas were scanned with a 50‐MHz frequency probe. The images were compared with those from normal skin at a contralateral site or at the lesion's margin. The ultrasonic images were collected by the same staff to avoid differences in the procedure.

### 2.3 Statistical analysis

Percentages of the classification data were used for statistical descriptions, and Fisher's exact test was used to determine whether there was significant difference in the ultrasonographic characteristics of the two diseases. A value of *P* < .05 indicated statistical significance. The sensitivity, specificity, positive predictive value, negative predictive value, positive likelihood ratio, negative likelihood ratio, and Jordan index were calculated. All statistics were processed by SPSS 20.0 software (IBM).

## 3 RESULTS

### 3.1 Ultrasonographic features of erythema in early‐stage PV

**Figure 1:**
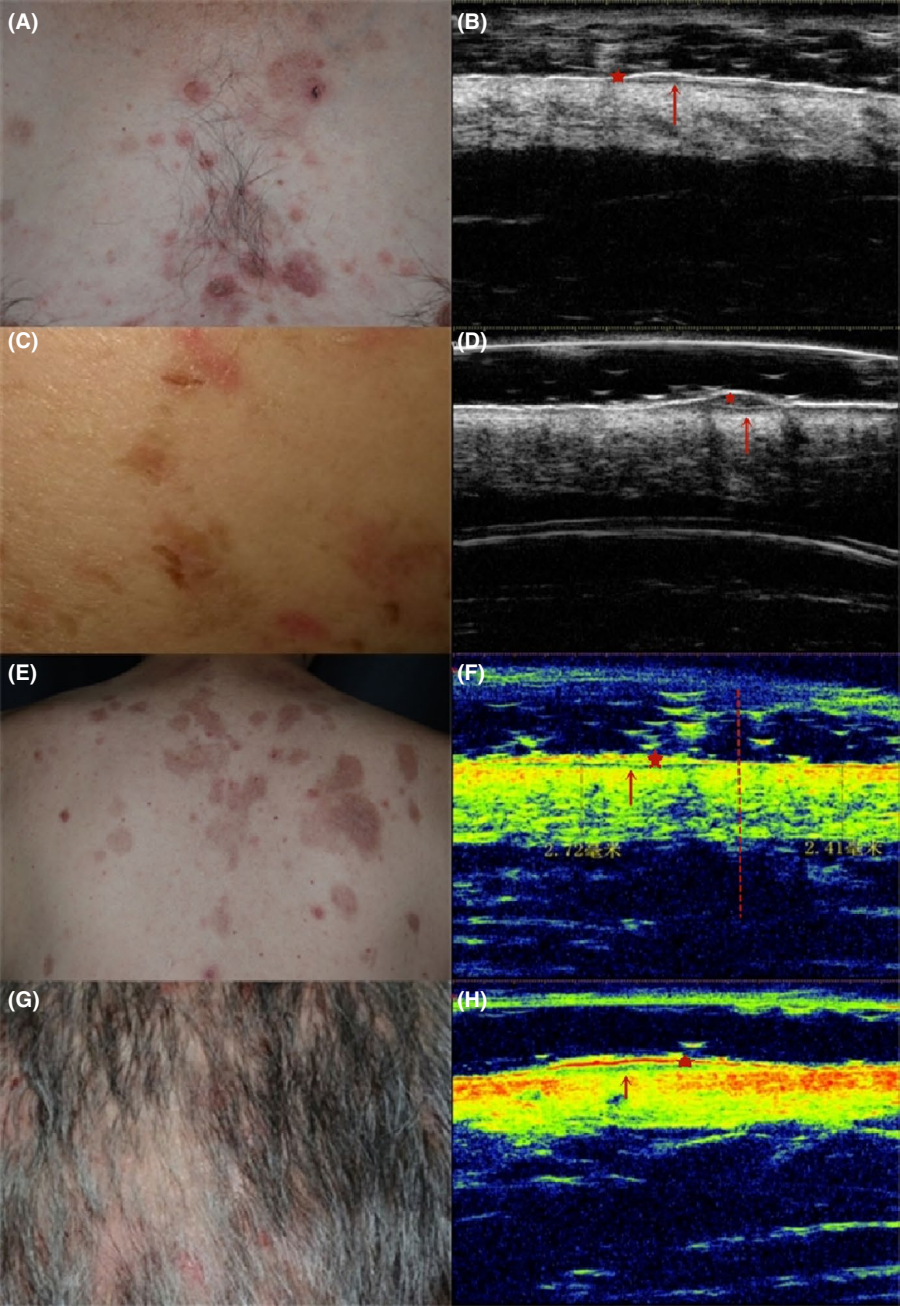
Clinical and ultrasonographic images of early‐stage PV patient. 1A, C, E, G, Macroscopic images. 1B, D, F, H, Corresponding ultrasonographic images. 1B, H, echo enhancement of epidermis and echo reduction of the dermis. 1B, D, F, H, linear or oval intra‐epidermal hypoechoic region (*), linear anechoic band at dermal‐epidermal junction (↑). 1B, 1D, 1F the thickness of the dermis is slightly increased

The ultrasonographic images of early‐stage PV patients (Figure 1, Table 1) showed enhanced epidermal echo (8/14, 57%), linear or oval intra‐epidermal hypoechoic or anechoic region (12/14, 86%), linear anechoic region at the dermal‐epidermal junction (14/14, 100%), reduced echo of superficial to whole dermis (9/14, 64%), and slightly increased dermal thickness (14/14, 100%). The PV patients’ features were significantly different from those of the normal control group (*P* < .05).

**Table 1:** Ultrasonographic characteristics of the erythema in patients with early‐stage pemphigus vulgaris compared with normal skin

Ultrasonic manifestation	Number of cases (n = 14[%])	*P* value
enhanced epidermal echo	8 (57)	.02
linear or oval intra‐epidermal hypoechoic or anechoic areas	12 (86)	<.01
Linear hypoechoic areas at the dermal‐epidermal junction	14 (100)	<.01
reduced echo of superficial to whole dermis	9 (64)	.01
slightly increased dermal thickness	14 (100)	<.01

### 3.2 Ultrasonographic features of erythema in SD

**Figure 2:**
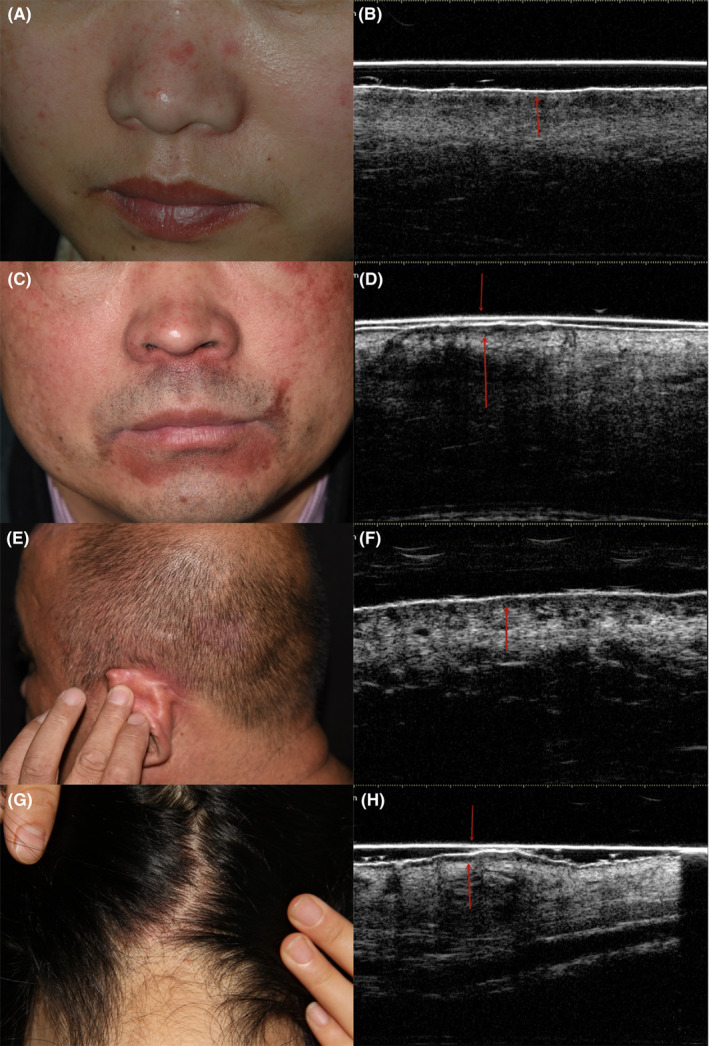
Clinical and ultrasonographic images of SD patient. 2A, 2C, 2E, 2G Macroscopic images. 2B, 2D, 2F, 2H Corresponding ultrasonographic images. 2D, 2H echo enhancement and unevenness of epidermis (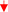), linear anechoic band at dermal‐epidermal junction (↑) and echo decrease of the middle dermis. 2B, 2F echo reduction of the superficial dermis (↑). 2B, 2D, 2F, 2H the thickness of the dermis is slightly increased

The ultrasonographic images of SD patients (Figure 2, Table 2) showed enhanced epidermal echo (7/14, 50%), epidermal unevenness (7/14, 50%), linear anechoic bands at the dermal‐epidermal junction (8/14, 57%), echo reduction of the middle dermis (10/14, 71%), and slightly increased dermal thickness (10/14, 71%). The SD patients’ images were significantly different from those of the normal control group (*P* < .05).

**Table 2:** Ultrasonographic characteristics of the erythema in patients with seborrheic dermatitis compared with normal skin

Ultrasonic manifestation	Number of cases (n = 14[%])	*P* value
enhanced epidermal echo	7 (50)	.06
epidermal unevenness	7 (50)	.06
linear anechoic band at dermal‐epidermal junction	8 (57)	.02
echo reduction of the middle dermis	10 (71)	<.01
slightly increased dermal thickness	10 (71)	<.01

### 3.3 Analysis of the differences and the diagnostic value of the ultrasonographic features

The VHFUS characteristics identified in the early‐stage PV patients were linear or oval intra‐epidermal hypoechoic or anechoic regions (86%), linear anechoic areas at the dermal‐epidermal junction (100%), and slightly increased dermal thickness (100%) (Table 3). The characteristic with highest specificity was the linear intra‐epidermal hypoechoic or anechoic bands (100%), which was significantly different from that in the SD patients (*P* < .05). In contrast, the SD patients’ most specific VHFUS erythematous characteristic of SD patients was uneven epidermis, but this characteristic was not significantly different between that found in the early‐stage PV patients (*P* > .05). Linear anechoic areas at the dermal‐epidermal junction were apparent in the erythematous areas of patients with early‐stage PV (100%) and those with SD (57%), although there was a significant difference between the two groups (*P* < .05).

**Table 3:** Ultrasonographic characteristics of the erythema in patients with early‐stage pemphigus vulgaris and those with seborrheic dermatitis

Ultrasonic manifestation	n = 14 (%)		*P* value	Sensitivity (%)		Specificity (%)		Positive predictive value (%)		Negative predictive value (%)		Positive likelihood ratio		Negative likelihood ratio		Jordan index	
	PV	SD		PV	SD	PV	SD	PV	SD	PV	SD	PV	SD	PV	SD	PV	SD
Enhanced epidermal echo	8 (57)	7 (50)	1	57	50	50	43	53	47	54	46	1.14	0.88	0.86	1.17	0.07	−0.07
Epidermal unevenness	0 (0)	7 (50)	.06	0	50	50	100	0	100	33	67	0	‐	2	0.5	−0.5	0.5
Linear or oval intra‐epidermal hypoechoic or anechoic region	12 (86)	0 (0)	<.01	86	0	100	14	100	0	88	13	‐	0	0.14	7	0.86	−0.43
Linear anechoic band at dermal‐epidermal junction	14 (100)	8 (57)	.016	100	57	43	0	64	36	100	0	1.75	0.67	0	‐	0.43	−0.43
Echo reduction of the dermis	9 (64)	10 (71)	1	64	71	29	36	47	53	44	56	0.9	1.11	1.25	0.8	−0.07	0.07
Slightly increased dermal thickness	14 (100)	10 (71)	.098	100	71	29	0	58	42	100	0	1.4	0.71	0	‐	0.29	−0.29

## 4 DISCUSSION

Ultrasonographic imaging technology was applied to skin thickness measurements during the late 1970s. In recent years, with continuous improvement in high‐frequency ultrasound technology, its application in the diagnosis of skin diseases is becoming increasingly widespread. High‐frequency ultrasound (HFUS) usually refers to ultrasound with a frequency of >10 MHz and VHFUS with a frequency of >30 MHz. The 50‐MHz ultrasound used in this study has high resolution and a penetration depth of 4 mm, exceeding the thickness of skin tissue. Therefore, it can be used to assess skin lesions. At present, high‐frequency ultrasound imaging is widely used to evaluate benign and malignant skin tumors, skin diseases with vascular abnormalities, nail diseases, and inflammatory diseases, and it is used in cosmetic dermatology.^3^, ^4^, ^5^, ^6^, ^7^ Additionally, the use of HFUS to evaluate inflammatory skin diseases is gradually being developed. It has been reported that HFUS can help to evaluate the severity and treatment efficacy of diseases such as scleroderma,^1^ psoriasis,^2^ and morphological changes in the nails of psoriatic patients.^8^ Its application in other inflammatory diseases, such as autoimmune blistering, panniculitis, and dermatomyositis, remains to be developed.

To date, there have been only two case reports on using VHFUS in PV patients, both of which focused on describing the ultrasound characteristics of typical blisters. Studies on VHFUS evaluation of erythema of early‐stage PV have not been reported. Xiujun Cheng et al^9^ performed VHFUS in two cases of PV, and the images of the blisters showed hyperechoic lines at the bottom of an anechoic area (ie, anechoic bands in the epidermis). We found that, for patients with early‐stage PV, ultrasonographic characteristics of the erythema were hypoechoic or anechoic areas in the epidermis and banded hypoechoic areas beneath the epidermis, consistent with the histopathological feature of intra‐epidermal fissures. Also, the size and shape of these hypoechoic or anechoic areas in the epidermis were closely related to the disease stage. In addition, VHFUS of the erythematous areas showed linear anechoic regions at the dermal‐epidermal junction, reduced echo of the superficial to whole dermis, and slightly increased dermal thickness, which was consistent with histopathological findings of inflammatory cell infiltration and vasodilation.

Reports on applying VHFUS to patients with SD are rare at present. We found that VHFUS of erythematous areas in SD patients revealed enhanced echo and unevenness of the epidermis, which histopathological evaluation showed was composed of a combination of scales, acanthosis, and follicular plugs. Histopathological assessment revealed linear anechoic bands at the dermal‐epidermal junction, echo reduction of the middle dermis, and slightly increased dermal thickness along with inflammatory infiltration and vasodilation.

Prior to the appearance of typical vesicles, bullae, and mucosal lesions, PV is often characterized by erythema, erosion, and crusting on the head, chest, and back during its early stage. It is easily misdiagnosed as SD, resulting in patients having missed the opportunity for early treatment. Although invasive examinations, such as histopathological assessment and immunofluorescence tests, during the early stage of the disease can reduce the misdiagnosis rate, they increase patients’ pain and financial burden. If VHFUS can be used to differentiate early‐stage PV and SD, it could reduce the misdiagnosis rate and decrease patients’ pain and financial burden.

Our study found that the ultrasonographic manifestations of early‐stage PV and SD have their own relatively specific characteristics. Linear or oval intra‐epidermal hypoechoic or anechoic areas comprised the most specific characteristic of early‐stage PV, with high sensitivity (86%). It was significantly different from SD, whose most specific characteristic was epidermal unevenness (100%), although the sensitivity was low (50%) and there was no significant difference from that of early‐stage PV. Thus, the VHFUS‐defined characteristic of linear or oval hypoechoic or anechoic areas in the epidermis can be used as a basis for differentiating early‐stage PV and SD. Epidermal unevenness can be used as a VHFUS feature to diagnose SD, but it cannot be used as the basis of a differential diagnosis. However, 100% of patients with early PV and only 57% of patients with SD in this study had the ultrasonic characteristic of a linear anechoic region at the dermal‐epidermal junction, and the difference between the two groups was significant.

## 5 CONCLUSION

VHFUS is an important noninvasive diagnostic tool that can assist in the differential diagnosis of early‐stage PV and SD, thereby reducing the number of clinical misdiagnoses. The application of HFUS in the diagnosis and differential diagnosis of more skin diseases requires further study.

## CONFLICT OF INTEREST

None reported.
